# Familiar white sponge nevus^☆^

**DOI:** 10.1016/j.abd.2023.08.013

**Published:** 2024-04-08

**Authors:** Camino Prada-García, Asunción González-Morán, Xenia Pérez-González

**Affiliations:** a Department of Dermatology, Complejo Asistencial Universitario de León, León, Spain; b Pathological Anatomy, Complejo Asistencial Universitario de León, León, Spain

Dear Editor,

White sponge nevus is a rare and benign disease with autosomal dominant inheritance and variable penetrance, primarily affecting the oral mucosa. It is found in approximately one in 200,000 people. This condition was initially described by Hyde in 1909 and presents as white or grey patches with a spongy appearance. These patches, typically located on the buccal mucosa bilaterally, do not peel off when scraped. They also appear on the lips, alveolar ridges, and the floor of the mouth.^1^, ^2^ We herein present two affected members of a single family.

A 61-year-old woman, with no significant medical or surgical history, sought consultation due to the presence of asymptomatic lesions in the oral mucosa that had been present since childhood. Examination revealed plaques with diffuse borders, that were non-infiltrated and with a whitish, velvety surface. These were located bilaterally on the buccal mucosa and the dorsal tongue. The lesions could not be removed by scraping and no other skin or mucosal lesions were observed (Fig. 1).

**Figure 1:**
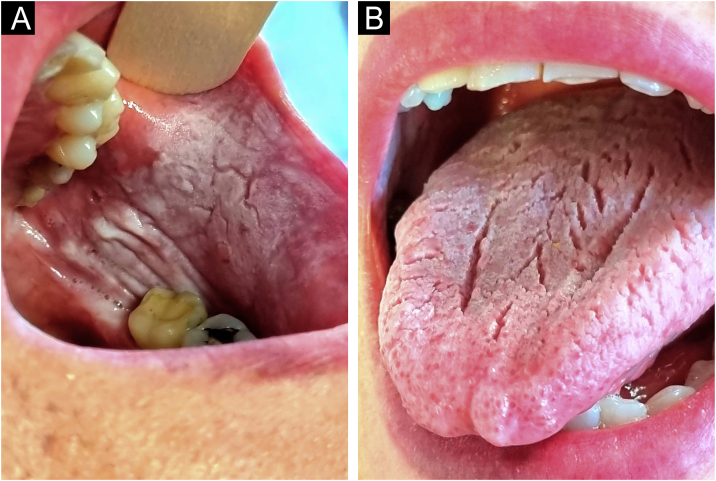
(A) Whitish lesions on the buccal mucosa. (B) Plaque with a whitish, velvety surface on the dorsal tongue.

Her 23-year-old son also sought consultation for the presence of similar, asymptomatic lesions that had been present since birth. The lesions were of the same characteristics, affecting the bilateral buccal mucosa and the dorsal tongue (Fig. 2).

**Figure 2:**
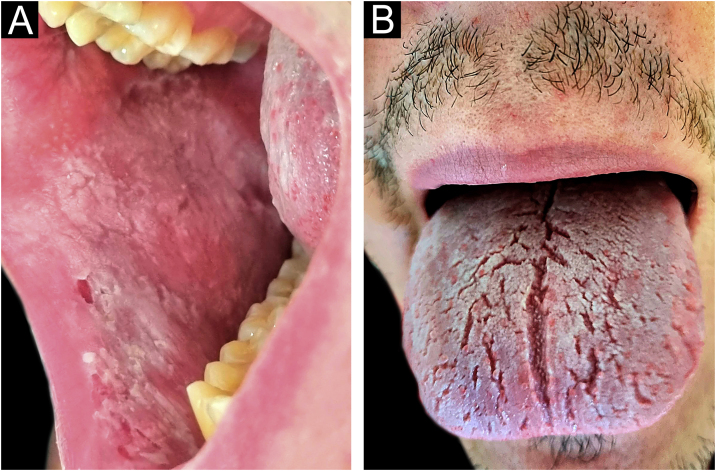
(A) Her son with a well-demarcated white patch present on the buccal mucosa. (B) Plaque with a whitish and a spongy appearance on the dorsal tongue.

A punch biopsy was performed on one of the lesions on the 61-year-old patient's buccal mucosa. It showed moderate acanthosis with regular elongation of crests and intracellular edema, alongside foci of parakeratosis and some dyskeratotic cells displaying paranuclear eosinophilic condensations. Only the upper layers were affected. In the underlying chorion, a few slightly dilated capillaries and a mild, nonspecific perivascular chronic infiltrate were observed. The Periodic Acid-Schiff (PAS) technique did not reveal any fungi (Fig. 3). Both clinical and histopathological findings were consistent with the diagnosis of familial white sponge nevus. Neither patient wished to receive any treatment as they had no symptoms or clinical complaints.

**Figure 3:**
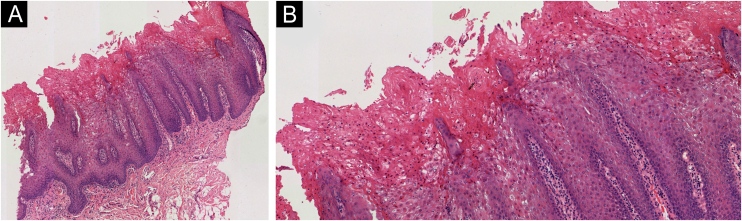
(A) Photomicrograph from Hematoxylin & eosin stained from the left buccal mucosa sample of the 61-year-old woman demonstrating moderate acanthosis with regular elongation of crests and intracellular edema (Hematoxylin & eosin, ×40). (B) Some dyskeratotic cells displaying paranuclear eosinophilic condensations (Hematoxylin & eosin, ×400).

White sponge nevus is a benign and rare keratinopathy, primarily affecting the oral mucosa. It was first described by Hyde in 1909 and Cannon coined the term “white sponge nevus” in 1935. Although it follows an autosomal dominant transmission pattern with incomplete penetrance, there have been reported cases without a family history.^1^, ^2^, ^3^ The most frequently associated mutations are found in the genes encoding Cytokeratins 4 (KRT4) and 13 (KRT13).^4^, ^5^ Lesions usually appear during childhood or adolescence without a preference for either sex. They present as asymptomatic white or greyish plaques, asymmetrical, with irregular borders and a spongy appearance, and are usually found bilaterally in the oral mucosa. Lesions may also appear on the nasal, esophageal, vaginal or rectal mucosae.^2^ Both cases in the present report had bilateral lesions on the buccal mucosa and on the dorsal tongue. The diagnosis can be made with a detailed patient history and meticulous clinical examination. A histopathological analysis may be performed purely to corroborate the initial diagnosis.^6^ The main histological findings include the presence of acanthosis with intercellular edema and vacuolization, as well as parakeratotic or orthokeratotic hyperkeratosis in the superficial layers. This entity may resemble a wide spectrum of oral disorders that may present as diffuse white plaques. The differential diagnosis should include oral candidiasis, frictional leukokeratosis, leukoedema, leukoplakia, lichen planus, and even squamous cell carcinoma. The manifestation of the lesions, including the plaque dimensions, the affected areas, and their distribution, may change with time. To date, no standard treatment exists, although various therapies such as topical and oral tetracyclines and chlorhexidine rinses have been tried with variable results. The beneficial effect of tetracycline may stem from the modulation of epithelial keratinization. Vitamins (like beta-carotene and topical applications of retinoic acid), antihistamines, and laser ablation have also been employed.^1^, ^3^, ^7^ Specific treatment is not generally required because this condition is benign and not expected to progress malignantly.

In conclusion, we describe two new cases of familial white sponge nevus, a rare entity that should be considered in the differential diagnosis of other “white” oral lesions with potential malignancy. Adults may display oral manifestations of white sponge nevus that clinically resemble other whitish oral lesions, which can lead to a delayed diagnosis based on microscopic findings. Early and accurate diagnosis of the condition is crucial to prevent the application of unnecessary treatments.

## Financial support

None declared.

## Authors’ contributions

Camino Prada-García: The study concept and design; data collection, analysis, and interpretation of data; writing of the manuscript or critical review of important intellectual content; effective participation in the research guidance; intellectual participation in the propaedeutic and/or therapeutic conduct of the studied cases; critical review of the literature; final approval of the final version of the manuscript.

Asunción González-Morán: The study concept and design, analysis and interpretation of data; writing of the manuscript or critical review of important intellectual content; intellectual participation in the propaedeutic and/or therapeutic conduct of the studied cases; critical review of the literature; final approval of the final version of the manuscript.

Xenia Pérez-González: The study concept and design; data collection, analysis, and interpretation of data; intellectual participation in the propaedeutic and/or therapeutic conduct of the studied cases; final approval of the final version of the manuscript.

## Conflicts of interest

None declared.
